# Rapid and gentle hydrogel encapsulation of living organisms enables long-term microscopy over multiple hours

**DOI:** 10.1038/s42003-018-0079-6

**Published:** 2018-06-21

**Authors:** Kyra Burnett, Eric Edsinger, Dirk R. Albrecht

**Affiliations:** 1 Department of Biomedical Engineering, Worcester Polytechnic Institute, 100 Institute Road, Worcester, MA 01609 USA; 2 Marine Biological Laboratory Josephine Bay Paul Center for Comparative Molecular Biology and Evolution, 7 MBL Street, Woods Hole, MA 02540 USA; 3 Department of Biology and Biotechnology, Worcester Polytechnic Institute, 100 Institute Road, Worcester, MA 01609 USA

## Abstract

Imaging living organisms at high spatial resolution requires effective and innocuous immobilization. Long-term imaging places further demands on sample mounting with minimal perturbation of the organism. Here we present a simple, inexpensive method for rapid encapsulation of small animals of any developmental stage within a photo-crosslinked polyethylene glycol (PEG) hydrogel, gently restricting movement within their confined spaces. Immobilized animals maintain their original morphology in a hydrated environment compatible with chemical treatment, optical stimulation, and light-sheet microscopy. We demonstrate prolonged three-dimensional imaging of neural responses in the nematode *Caenorhabditis elegans*, recovery of viable organisms after 24 h, and imaging of larger squid hatchlings. We characterize a range of hydrogel and illumination conditions for immobilization quality, and identify paralytic-free conditions suitable for high-resolution single-cell imaging. Overall, PEG hydrogel encapsulation provides fast, versatile, and gentle mounting of small living organisms, from yeast to zebrafish, for continuous observation over hours.

## Abstract (web summary)

Kyra Burnett et al. present a simple and economical method to encapsulate small living organisms for long-term microscopy in a photo-crosslinked polyethylene glycol hydrogel. This method provides a fast and gentle mounting for continuous imaging over hours, and works with light-sheet microscopy and optogenetic stimulation.

## Introduction

Fluorescence microscopy has had a profound impact on biomedical research, providing spatial and temporal information about gene expression, molecular dynamics, morphology of labeled structures^1^, and functions such as genetically encoded calcium indicators that indicate activity of electrically excitable cells^2^. While many imaging experiments last only minutes per sample, long-term time-lapse microscopy can indicate changes in more gradual processes lasting hours or days. These longitudinal studies require reliable immobilization of the sample during imaging periods while simultaneously preserving the organism’s health and maintaining the function of fluorescent markers. Thus, methods of immobilization must be compatible with required environmental conditions such as hydration, temperature, and nutrition, and with imaging parameters for maximal signal with minimal phototoxicity and sample perturbation^3^.

Small model organisms are widely used for in vivo studies of basic physiology and systemic responses. The nematode *Caenorhabditis elegans* is a particularly useful model organism due to its <1 mm size, optical transparency, short life cycle, and ease of genetic manipulation. Standard methods for immobilization of *C. elegans* include treating the organism with paralyzing reagents such as the mitochondrial inhibitor sodium azide or the acetylcholine agonists tetramisole or levamisole^4^. However, chemical paralytics have disadvantages: azide causes gradual fluorophore bleaching, whereas tetramisole contracts the body, altering some physical structures. Additionally, these anesthetic reagents have toxic effects on the worm when applied during long-term studies. Another method mechanically immobilizes worms with nanobeads^5^ and is advantageous for studies in which recovery and long-term animal health post imaging are needed, such as after laser axotomy or laser cell ablation^6^. In both chemical and nanobead immobilization, the animal remains in a closed environment sandwiched between a coverslip and an agar pad throughout the duration of the experiment, preventing the application of external probes (such as a microinjection needle) or chemical stimuli for neurosensory or physiological studies. Further, the closed environment limits gas exchange and leads to hypoxic conditions within minutes^7^. For worm immobilization in physically accessible and open environments, animals have been glued for electrophysiology^8^ or mounted under paraffin oil for microinjection^9^. In both cases, animal health can deteriorate over time and the immobilization methods are not compatible with long-term studies.

Microfluidic devices can trap animals in small confined geometries for neural recordings and ablations^10, 11^, parallel animal imaging^12^, and worm sorting applications^13, 14^. Some have the ability to present chemical stimuli quickly and precisely^10, 15^, and most use gas-permeable materials that maintain suitable oxygenation. Microfluidic systems can also reversibly immobilize by pneumatic compression^11, 16^, CO_2_ exposure^17^, or cooling^14^. However, these devices take substantial time and resources to fabricate and set up and are sensitive to operational issues including fluidic leaks and clogs. Therefore, less complex methods may be preferred when microfluidic advantages such as dynamic trapping are unnecessary.

An alternative approach to immobilization encapsulates the sample in a three-dimensional (3D) hydrogel. Low-melting-point agarose is used to immobilize larger samples such as zebrafish^18^, but its relative softness allows smaller organisms to move and burrow. A thermoreversible Pluronic hydrogel can allow periodic cycles of *C. elegans* immobilization and release^19^, gelling at 25 °C and solubilizing upon cooling to 22 °C. These hydrogels are also soft, gelation is slow, and precise temperature control is required, making them challenging to use for continuous long-term, high-resolution imaging. Alternatively, covalently crosslinked hydrogels, such as those based on poly(ethylene-glycol) (PEG), can maintain permanent stiffness and form a gel quickly at any temperature. PEG hydrogels have been studied extensively for cell culture and other applications^20–22^ and can embed cells for long periods up to several weeks^23, 24^. PEG hydrogels are particularly attractive biomaterials for their tunable mechanical, diffusive, and optical properties that can be varied easily by monomer chain length and concentration^25–27^.

Here, we explore the use of PEG hydrogels as an embedding medium for continuous long-term imaging. In particular, we describe a rapid, convenient method for mounting and immobilizing small organisms that gently encapsulates them in a non-toxic, photosensitive PEG hydrogel, trapping them within a confined volume. The covalently crosslinked hydrogel immobilizes organisms within seconds of light exposure, holds them permanently for long-term studies, yet can be easily broken to release them. The encapsulation process uses standard lab equipment and readily available materials, works with any size organisms, and can occur at any desired temperature, unlike thermally gelling hydrogels that require heating or cooling. The method is versatile, compatible with a wide range of hydrogel size, stiffness, and diffusivity, and the degree of animal constraint can be controlled. Here, we characterize the speed and quality of immobilization for high-resolution imaging under varying conditions including light sources, substrates, polymer concentrations, and buffer conditions. We focus our characterization on *C. elegans* as a particularly challenging organism for immobilization, as it is highly motile, relatively strong for its size, and capable of burrowing. We also demonstrate hydrogel encapsulation for larger, softer marine organisms such as pygmy squid hatchlings.

Additionally, we show here that PEG hydrogel encapsulation is well suited for light-sheet fluorescence microscopy (LSFM), an attractive imaging modality for continuous long-term 3D imaging because it reduces photobleaching by more than an order of magnitude compared with confocal systems^28^. LSFM has enabled monitoring of embryo development over several hours in liquid-filled open-top chambers^29–31^. However, diffraction-limited optical imaging occurs only when both the excitation light sheet and the emitted fluorescence pass through refractive index-matched materials, making LSFM challenging to use with standard methods for mounting larval and adult animals^31, 32^, but convenient to use with hydrogels that are nearly index-matched with water.

Overall, PEG hydrogel encapsulation is a rapid, gentle, versatile, and inexpensive alternative mounting method useful for continuous long-term imaging, in an open format that may benefit other *C. elegans* techniques such as laser ablation and microinjection, and scalable to other model organisms from yeast to *Drosophila* and zebrafish.

## Results

### Immobilization of *C. elegans* within PEG hydrogels

**Fig. 1:**
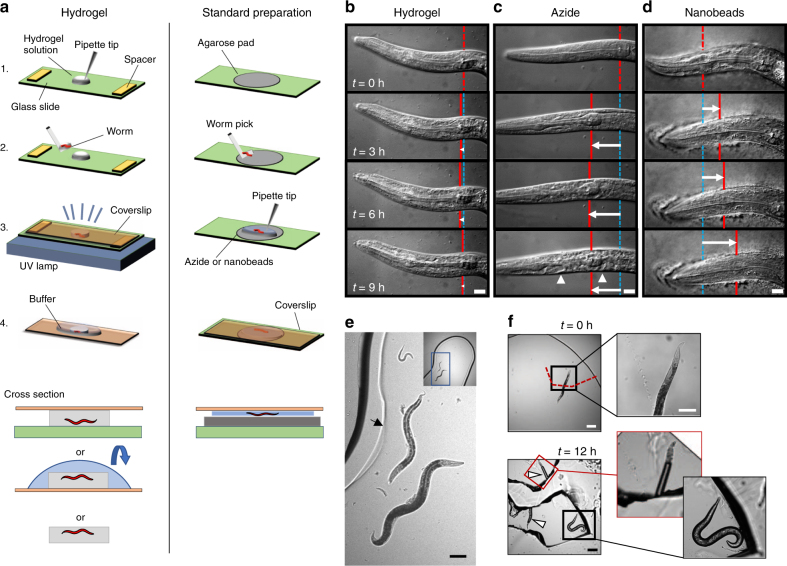
Mounting live *C. elegans* in hydrogels and on agarose pads for microscopy. **a** Schematic of worm mounting procedures by PEG hydrogel encapsulation or on agar pads. **b**–**d** Images taken over 9 h in a 10% PEG hydrogel (**b**) or on agarose pads with 25 mM sodium azide (**c**) or with 100 nm polystyrene beads (**d**). Red vertical line indicates a pharyngeal landmark position at each time point, blue lines indicate the initial landmark position, and white arrow indicates displacement relative to time *t* = 0 h. Arrowheads indicate morphological changes such as necrotic cell death after prolonged azide exposure. Scale bar, 10 µm. **e** Multiple larval and adult stages embedded in the same hydrogel. The hydrogel edge is indicated by a faint line (arrow), surrounded by about 100–200 µm of uncrosslinked polymer. Scale bar, 100 µm. **f** Worms embedded in a 20% PEG hydrogel were imaged immediately after crosslinking and after release 12 h later. Arrowheads indicate cavities in the hydrogel formerly occupied by worms. Scale bars, 200 and 100 µm (inset)

Encapsulation of animals in a PEG hydrogel comprises the following steps: preparing a glass slide base and cover, placing spacers that determine hydrogel geometry onto the glass slide base, pipetting the hydrogel precursor solution, picking animals into the precursor droplet, covering the droplet with a coverslip, and crosslinking the hydrogel by brief exposure to light (Fig. 1a). During light exposure, the hydrogel increases viscosity until gelation up to the surface of the embedded worm (Supplementary Movie 1). Hydrogel crosslinking occurred within seconds and immobilization could last for days, as animals could not escape or move beyond their encapsulated space (Fig. 1b). Some micron-scale movement was observed within this confined space during contraction of body wall muscles, as animals occasionally pushed against the flexible hydrogel, although this movement could be controlled with different preparations (see “Tunable immobilization of *C. elegans* by buffer conditions” below, and Supplementary Movie 2).

By comparison, conventional worm mounting requires melting agarose into a thin pad, picking animals onto the pad, adding a chemical or mechanical immobilizer, and covering with a coverslip (Fig. 1a). Animals exposed to 25 mM sodium azide, which inhibits mitochondrial function and relaxes muscle tone, can take an hour or more to fully immobilize (Fig. 1c). Animals immobilized more quickly with 100 nm polystyrene nanobeads than with azide, but they could still move gradually over hours (Fig. 1d).

Differential interference contrast (DIC) images at ×100 magnification showed minimal change in cellular morphology in animals embedded in a 10% PEG hydrogel (Fig. 1b–d). In contrast, sodium azide-paralyzed animals displayed characteristics of necrotic cell death after 6 h^33^. Nanobead immobilization is effective only with compression, causing an apparent increase the width of the worm^5^. Overall, worms encapsulated in the hydrogel over 9 h showed fewer physical alterations compared to azide and nanobead preparations.

Animals of all stages, from eggs to larval stages to adults, could be mounted in the same hydrogel (Fig. 1e). Encapsulated animals were recoverable by breaking the hydrogel with gentle pressure from a worm pick or fine-point forceps (Fig. 1f). After 24 h of hydrogel encapsulation, young adult *C. elegans* remained mostly viable with 86% (207/241) able to crawl away upon release. The nonviable animals appeared physically damaged, from forceps during release or internal egg hatching, suggesting that hydrogel encapsulation per se was not directly responsible for loss of viability.

### Parameters affecting PEG hydrogel crosslinking

**Fig. 2:**
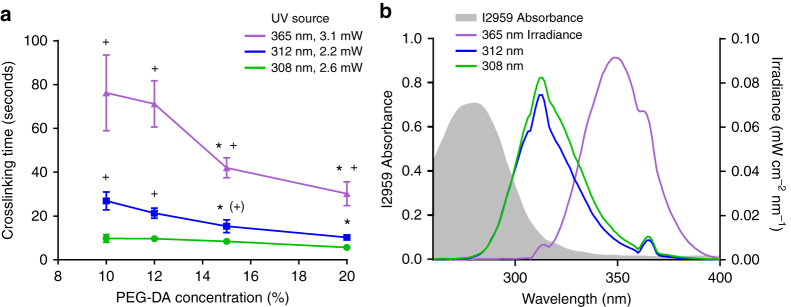
Characterization of PEG hydrogel crosslinking rates. **a** Crosslinking time determined by immobilization of young adult animals in 10–20% PEG-DA exposed with 365, 312, and 308 nm ultraviolet sources. Each point represents the mean of *n* = 10 independent trials, with each trial averaging measurements from two to five worms. Error bars represent standard deviation. Statistics were performed using ordinary two-way ANOVA with Bonferroni’s post hoc tests for pairwise comparisons: *P < 0.001 for each concentration compared to 10% PEG-DA, and ^+^*P* < 0.001 or ^(+)^*P* < 0.05 for each source compared to 308 nm UV. **b** Absorbance spectrum of 0.001% I2959 (gray, left axis), and emission spectra for each ultraviolet exposure source (right axis). Emission spectra were normalized such that area under each curve matched total output power

The mechanical, diffusive, and optical properties of PEG hydrogels can be tuned via monomer chain length and concentration^25–27^. To determine how gelation properties are affected by polymer concentration and other photocrosslinking parameters, we imaged animal movement during crosslinking and measured the exposure time at which motion ceased. Using several ultraviolet (UV) light sources, hydrogels of different size, monomer concentration, and photoinitiator concentration gelled in <1 min with an irradiance dose of 15–220 mJ cm^−2^ (Fig. 2a). Higher concentrations of PEG-DA reduced the required time of UV light exposure, with a 20% hydrogel gelling in about one-half the exposure time required for a 10% hydrogel.

The intensity and wavelength of light exposure affects crosslinking rate. The absorbance of the I2959 photoinitiator drops rapidly above 300 nm (Fig. 2b) such that shorter wavelength UV sources crosslink the hydrogel more efficiently. For example, a 308 nm medium-wave UV-B transilluminator box gelled the polymer in 6–10 s at 2.6 mW cm^−2^ (16–26 mJ cm^−2^ dose) for 20–10% hydrogel concentration, due to strong overlap between light emission and photoinitiator absorbance. Similarly, a 312 nm handheld medium-wave UV-B light required 12–25 s exposure at 2.2 mW cm^−2^ (30–55 mJ cm^−2^ dose). A 365 nm long-wave UV-A source required a longer exposure time of 30–70 s at 3.1 mW cm^−2^ (95–220 mJ cm^−2^ dose) for 20–10% polymer concentration due to weak photoinitiator absorbance at this wavelength. Glass slides and cover slips that absorb strongly at UV-B wavelengths (Supplementary Fig. 1), such as those made from soda-lime glass and many plastics, require extended exposure times.

Hydrogel geometry had a minor effect on crosslinking rates. Thinner hydrogels (100 vs. 500 µm) and smaller hydrogels (1 vs. 10 µL) gelled slightly faster by <20% (Supplementary Fig. 2). Photoinitiator concentration did not affect crosslinking rate.

### Tunable immobilization of *C. elegans* by buffer conditions

**Fig. 3:**
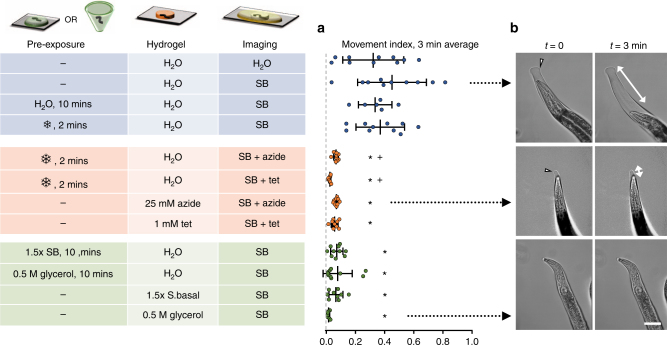
Buffer conditions before, during, and after hydrogel crosslinking influence immobilization for microscopy. Pre-exposure to hypo- or hyper-osmotic solutions for 10 min, or cooling pretreatment (snowflake symbol represents on ice or in a –20 °C freezer for 2 min), occurred in a droplet or microtube. Hydrogel solutions (20%) were prepared in water (H_2_O), S-Basal buffer (SB), 25 mM sodium azide (azide) or 1 mM tetramisole (tet) in water, 500 mM glycerol in water, or 1.5× S-Basal buffer. **a** Each dot represents the mean movement index over 3 min (Supplementary Figs. 4, 5), *n* *=* 7–10 worms per condition. Vertical and error bars represent mean and standard deviation. **b** Images represent typical movement under the conditions indicated by black arrows. Arrowheads indicate the edge of the hydrogel; animal movement can occur within this confined space (white arrows). Scale bar, 30 μm. Statistics were performed using ordinary two-way ANOVA with Bonferroni’s post hoc tests for pairwise comparisons: *P < 0.0001 compared with the hydrogel control with SB, and ^+^*P* < 0.0001 compared with the hydrogel cooling control with SB

Because encapsulated animals could push against the hydrogel and move or rotate slightly, we explored modifications that would reduce micron-scale movement for long-term high-resolution microscopy (Fig. 3). Micron-scale movement was quantified by tracking nuclei over 3 min (Supplementary Fig. 3) and by an image-based movement index (MI) sensitive to changes in position, rotation, focus, and photobleaching (Supplementary Fig. 4). The range of movement of animals embedded in 20% PEG hydrogels in water averaged 39 μm over 3 min, with an average MI of 0.33 ± 0.21. Exposure to the paralytic reagents sodium azide or tetramisole reduced micron-scale motion, both when applied during gelation and when applied transiently after encapsulation (18 µm, MI = 0.07 ± 0.01, and 19 µm, MI = 0.06 ± 0.02, respectively; *P* < 0.0001). Photobleaching observed when using sodium azide contributed a majority of the MI compared with tetramisole (Supplementary Fig. 5).

Toward paralytic-free tight immobilization, we explored the ability for cooling temperatures or osmotic changes to reduce movement. Cooling to about 4 °C on ice temporarily immobilizes animals^14^. However, while cooling stopped thrashing before crosslinking, movement after embedding remained similar to uncooled animals. Cooling also allowed animals to settle within the hydrogel droplet, positioning them parallel to the glass substrate for improved imaging.

Buffer osmolarity changes body size, shrinking animals in hyper-osmotic solutions over the course of minutes through the loss of water^34^. We reasoned that animals placed in a hyper-osmotic solution before or during gelation would shrink, thereby reducing the encapsulation space and tightening their confinement upon return to normal osmolarity and body size. Conversely, swelling animals before crosslinking in hypo-osmotic solutions could expand the hydrogel space, thereby providing more space for movement. However, crosslinking the hydrogel in water (0 mOsm), then imaging in S-Basal buffer (280 mOsm), did not significantly increase movement (39 ± 17 µm range over 3 min, MI = 0.45 ± 0.24) compared with imaging in water (41 ± 28 µm, MI = 0.33 ± 0.21). In contrast, animals in a hyper-osmotic solution of 0.5 M glycerol (500 mOsm) or 1.5× S-Basal buffer (420 mOsm) for 10 min prior to or during crosslinking, then imaged in normal osmolarity S-Basal buffer, were well immobilized (8 ± 7 µm range, MI = 0.02 ± 0.007 and 15 ± 9 µm range, MI *=* 0.07 ± 0.05, respectively). These movement levels were comparable to immobilization by chemical paralytics.

### Light-sheet imaging of neural activity in unparalyzed worms

**Fig. 4:**
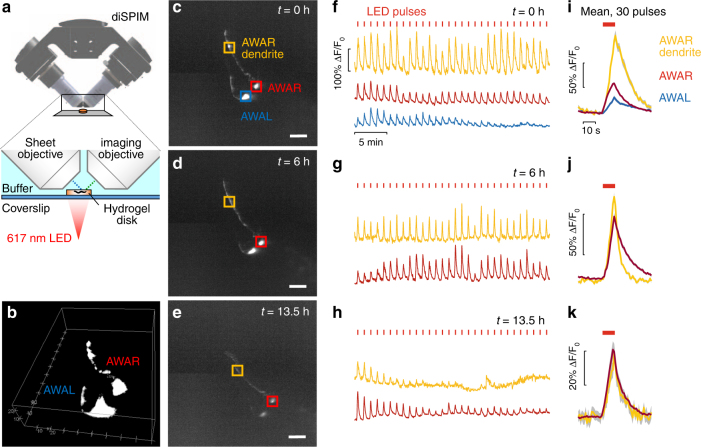
Long-term volumetric imaging of optogenetically stimulated neurons in living *C. elegans*. Animals expressing Chrimson and GCaMP2.2b in AWA sensory neurons were stimulated by red light and imaged using a diSPIM light-sheet microscope. **a** Schematic of diSPIM objectives, hydrogel, and red light exposure. **b** Three-dimensional volumetric view of AWA neurons. **c**–**e** Maximum intensity projections highlighting ROIs of AWAL and AWR cell bodies and AWAR dendrite, at *t* = 0 h (**c**), 6 h (**d**), and 13.5 h (**e**) time points. Scale bars, 20 μm. **f**–**h** Time-lapse recordings of GCaMP signal in each ROI beginning at each time point. The stimulation LED was pulsed for 10 s, once per minute. Continuous recordings lasted for 1 h per time point; here, 30 min are shown for clarity. **i**–**k** Mean fluorescence (Δ*F*/*F*_0_) is calculated for the 30 stimulation pulses indicated in **f**–**h**, with shading indicating s.e.m., and red bars above indicating 10-s red light exposure

Light-sheet microscopy with water-immersion objectives requires samples embedded within a medium index-matched with water, typically the hydrogel agarose. PEG hydrogels compare favorably with the standard 1% low-melt agarose (LMA) used in light-sheet protocols, with slight improvements in resolution and lower autofluorescence (Supplementary Fig. 6 and Supplementary Fig. 7). We tested PEG hydrogel immobilization for long-term neural imaging in young adult *C. elegans* without chemical paralytics, by hyper-osmotic preconditioning with 500 mM glycerol during crosslinking. Worms expressing the Chrimson light-sensitive ion channel and GCaMP calcium reporter in the AWA neuron pair were imaged for three 1 h trials spanning 14.5 h, stimulated every minute with a 10 s pulse of red light. Responses were reliably observed in the soma and neurites of both AWAL and AWAR neurons after embedding, and continued strongly in AWAR after 6 and 13.5 h at room temperature (Fig. 4). Normalized AWAR response magnitude declined about two-fold over 14 h in the cell body and five-fold in neurites, reminiscent of adaptation seen in chemical stimulation of these neurons^15^. Responses in AWAL declined more strongly over the first hour, but were still observed at the beginning of 6 and 13.5 h trials. Moderate photobleaching, which contributed an estimated 40% and 60% decline in baseline fluorescence at 6 and 13.5 h, respectively, somewhat reduced signal-to-noise ratio while maintaining the ability to observe long-term neural response dynamics.

### Encapsulation and imaging of squid hatchlings

**Fig. 5:**
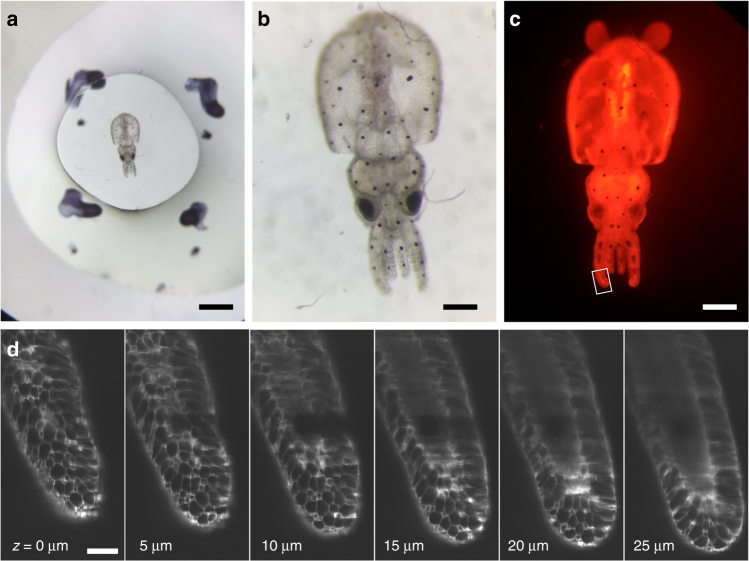
Encapsulation of a living pygmy squid hatchling in PEG hydrogel for light-sheet imaging. **a** Three-day-old squid hatchling was stained with 1 µM BODIPY C3 succinimidyl ester and embedded in a 1.2 mm thick, 4 mm diameter hydrogel disk and viewed in brightfield (**a**, **b**) and on a fluorescent dissecting microscope (**c**). **d** Raw light-sheet image slices of one squid arm indicated in panel **c** (box) are shown at 5 µm increments. Scale bars: 1 mm (**a**), 250 µm (**b**, **c**), 50 µm (**d**)

Soft mounting methods are especially important for flexible specimens, such as marine organisms. We encapsulated 3-day-old pygmy squid hatchlings in 1.2 mm thick, 4 mm diameter 16% PEG hydrogel disks under mild anesthesia (for orientation and positioning) and transferred them to seawater (Fig. 5). Within minutes of anesthetic washout, chromatophores actively opened and closed, indicating recovery of muscular function in internal structures. External structures in contact with the hydrogel, including arms, fins, and mantle, were immobilized. Prior to encapsulation, squid were stained with 1 µM BODIPY C3 succinimidyl ester dye to generally label cell boundaries throughout the organism (Fig. 5c). A volumetric stack was obtained using the diSPIM light-sheet microscope through the tip of one arm of the squid (Fig. 5d). Sharp cellular borders and slice alignment indicate minimal movement during image capture.

## Discussion

We present here a method for gentle, effective immobilization of living organisms for long periods of time in covalently photo-crosslinked PEG hydrogels. Mounting larval and adult *C. elegans* by hydrogel encapsulation was as simple as, or potentially easier than, standard agarose preparations, and offers several advantages. The hydrogel material is inexpensive ($10 per mL or 1¢ per µL) and photocrosslinking equipment is already present in most biological labs. Animals were immobilized faster than chemical paralytics, more completely than with nanobeads, and without compression or hypoxia as can occur under cover glass. The overall encapsulation process including all setup time takes only minutes, with gelation occurring within seconds. The amount of organism movement within the hydrogel cavity could be tuned by applying different solutions that rapidly diffused into the hydrogel. For example, transient exposure to standard paralytic chemicals restricted micron-scale movements within minutes. Alternatively, we identified a paralytic-free immobilization method that temporarily reduced worm body size in hyper-osmotic solutions before crosslinking, thereby tightening the hydrogel and immobilizing the animal as effectively as chemical paralytics. High-resolution image quality was maintained over multiple hours of recording in PEG hydrogels, and most animals were recoverable even 24 h later by physically breaking the hydrogels and allowing animals to escape from their confined spaces.

PEG hydrogels can be crosslinked in various sizes, making this approach suitable for embedding biological samples from <1 µm to mm or larger, a size range spanning from bacteria, yeast, and mammalian cells to small organisms including nematodes, marine organisms, *Drosophila*, and zebrafish. We demonstrated encapsulation of living pygmy squid hatchlings, ~1.5 mm long, whose hearts beat internally while soft motile external structures such as arms, fins, and mantle remained immobilized for light sheet or confocal microscopy.

The hydrogel mounting method is versatile and compatible with various polymer concentrations that tune mechanical stiffness and diffusive properties. Mechanical stiffness increases 10-fold from 30 to 300 kPa between 10 and 20% (w/v) concentrations of PEG-diacrylate (PEG-DA, 3 kDa), whereas mesh size decreases from 4 to 3 nm over this range^35^. In these hydrogels, small molecules can diffuse freely, but larger proteins cannot. Longer PEG-DA monomer chains can extend pore size over 10 nm, enabling diffusion of small proteins, while still immobilizing animals. Bacterial food would not permeate the hydrogel, although embedded animals in principle could be nourished by a chemically defined medium such as *C. elegans* Maintenance Medium^36^.

Higher PEG-DA concentration reduced crosslinking time, as reported previously^37^, and stiffer hydrogel disks were easier to handle and transfer than weaker ones. However, animal immobilization was as good in 12% gels as in stiffer 20% gels (Supplementary Fig. 8). This suggests that animals are unable to move once a threshold stiffness is achieved, allowing the user to select a concentration optimal for diffusion or handling properties. For example, a concentration of 12% may be optimal for worm immobilization with higher diffusivity, when gels need not be moved, while higher concentrations may be preferred if gels need to be handled manually.

PEG-DA gelation involves activation of the photoinitiator and covalent joining of acrylate polymer chain end-groups via radical chemistry. Oxygen quenches the crosslinking reaction by scavenging the initiator radicals, slowing or preventing gelation. Thus, PEG-DA solutions exposed to air do not gel, and hydrogel disks formed between glass slides retain a ~100 µm border of ungelled polymer where exposed to air (Fig. 1e). This effect may explain subtle increases in exposure requirements for taller and larger hydrogel disks, which have a larger surface exposed to air. Purging the surrounding space with an inert gas, such as nitrogen, effectively reduces this ungelled border, although this step is generally unnecessary unless a precise hydrogel geometry is needed.

PEG hydrogels can be crosslinked with numerous photoinitiators sensitive to ultraviolet or visible light. Most visible light photoinitators are either insoluble, cytotoxic, or slow to gel^38, 39^, although lithium phenyl-2,4,6-trimethylbenzoylphosphinate (LAP) is a good blue-light-activated alternative^40^. Here, we chose Irgacure 2959, a UV-sensitive initiator, for its rapid crosslinking time and extensive studies showing compatibility with cell culture^22, 23^. The UV-B irradiance used here at 2–3 mW cm^−2^ is comparable to sunlight and, in fact, PEG hydrogels can be crosslinked with sun exposure alone. The exposure dose for PEG-DA crosslinking, below 100 mJ cm^−2^, is two orders of magnitude below levels reported to induce lethality^41^ (~15 J cm^−2^), and animals remained viable following exposure and encapsulation. However, thresholds for behavioral effects are much lower. Wild-type animals can directly sense blue and UV light via the LITE-1 channel and respond to light exposures comparable to photoencapsulation with altered locomotion speed^42^. These responses generally subside within minutes, although the duration of any light-related behavioral effects should be evaluated for new assays.

Brief exposure to photoinitiator-free radicals could also cause oxidative damage. Animals remained mostly viable even after 24 h of encapsulation, including ultraviolet and radical exposure during crosslinking, suggesting minimal physiological perturbation, although specific assays for oxidative stress were not performed here. In cell culture, pre-incubation with antioxidants such as ascorbate reduced effects of free radicals on cell viability^43^ without affecting hydrogel crosslinking; thus, any oxidative stress noted in *C. elegans* could potentially be mitigated by similar pre-exposure to antioxidants[44].

PEG hydrogels could be crosslinked by a variety of UV-A and UV-B sources, many already available in biological labs for DNA gel documentation, including handheld lamps, transilluminator boxes, flashlights, and sunlight. Gelation time is dependent on the amount of light absorbed by the photoinitiator: shorter-wave UV-B sources (308, 312 nm) crosslinked faster as they better match the photoinitiator absorbance compared with UV-A sources (365 nm). A narrow-range LED flashlight (365 nm) could also crosslink the hydrogel, but required over 6 min of exposure due to minimal spectral overlap. Shorter-wave UV can be blocked by different glass materials (Supplementary Fig. 1). For example, UV-C lamps (254 nm), should overlap well with I2959 absorbance, but did not cause gelation as most glass and cover slips absorb nearly all ultraviolet light in this band. With UV-B sources, we found substrates composed of soda-lime glass required about twice the exposure time as borosilicate glass.

For time-lapse microscopy, embedded animals were easily identifiable across time points, as they could not escape. However, they could still move within their encapsulation volume more than might be desired for high-resolution microscopy. Paralytics could diffuse into the hydrogel, limiting movement to <8 μm over 3 min. Altering osmolarity before crosslinking could immobilize a worm to the same level without paralytics. While long-term exposure to high osmotic conditions negatively impacts animal health^34^, the physiological consequences of transient hyper-osmotic exposure over minutes remain to be studied. Overall, the choice to immobilize by osmotic or chemical methods will depend on the biological process under investigation. For example, neural studies in which fluorescent intensity of calcium reporters is the readout are strongly impacted by the use of the paralytic 2,3-butanedione monoxime, which inhibits Type II myosin but also affects calcium channels^45^, and sodium azide, which causes steady photobleaching over tens of minutes (Supplementary Fig. 5). Conversely, the acetylcholine agonist tetramisole contracts muscle and alters body shape, making it suitable for neural studies but perhaps not for morphological readouts. While immobilization itself plays little role in some biological functions, such as sensory neural responses^15^, others that are influenced by body movement, such as neuromuscular activity and organismal development, may need methods that allow periodic movement between imaging sessions^16, 19^. Outside of these situations, we propose that our hydrogel encapsulation method, with hyper-osmotic pre-exposure, may serve a wide variety of imaging modalities and biological applications.

LSFM can record the same sample for hours, such as the entirety of *C. elegans* embryo development^28^, because it requires less excitation light and reduces photobleaching and phototoxicity. However, LSFM requires that both the excitation light and the emitted fluorescence pass through refractive index-matched materials, complicating its use with standard *C. elegans* mounting methods^31^. Most LSFM protocols embed samples in low melting point agarose, which has good optical properties and immobilizes larger organisms well, while smaller organisms move substantially. We show here that PEG hydrogels restrict animal movement well, and both autofluorescence and imaging resolution are similar to or slightly better than agarose (Supplementary Fig. 6 and Supplementary Fig. 7). As a demonstration of long-term 3D microscopy, unparalyzed young adult animals were embedded in PEG hydrogels and imaged in a dual-inverted selective plane illumination microscope (diSPIM) for over 14 h. Reliable calcium transients were recorded in the soma and processes of the AWA chemosensory neurons expressing the calcium sensor GCaMP, triggered by optogenetic red-light activation of Chrimson cation channels. We observed no autofluorescence of the hydrogel itself. Neural response magnitude declined over hours, as is expected during sensory neural adaptation, yet overall animal viability was maintained and changes in functional reporters were observable over time scales that span state changes, which may last several hours. Some photobleaching occurred during hours of light-sheet exposure, somewhat reducing the signal-to-noise ratio, but at a rate far lower than alternate imaging methods. For example, photobleaching typically limits spinning-disk confocal microscopy (SDCM) recordings to about 15–30 min^46–48^, due to its requirement for far greater excitation intensity than LSFM. SDCM is also difficult to use with simultaneous optogenetic activation and optical readout of calcium activity, due to partial spectral overlap between light-sensitive channel and calcium sensor excitation wavelengths (at least with current sensor/channel pairs such as GCaMP/Chrimson and RCaMP/ChR2). Previously, no methods could restrain hatched *C. elegans* under the open environmental conditions required by the diSPIM, whereas embryos could be directly attached to the substrate^49^. Here, by physically preventing thrashing in larval and adult worms, the hydrogel encapsulation method allows the study of post-embryonic processes in a light-sheet system, and to our knowledge it represents the only current 3D neural imaging method compatible with long-term optogenetic stimulation in adults.

Overall, this method provides researchers with a gentle, rapid, inexpensive way to immobilize *C. elegans* and other organisms for continuous long-term experiments up to several hours, as a complement to existing sample mounting methods. It may not be suitable for experiments in which feeding is required, or for experiments requiring full movement between imaging periods. Nonetheless, by providing open fluidic access to the hydrogel and the potential for paralytic-free imaging, this method benefits long-term studies of dynamic processes in *C. elegans* and other small model organisms, such as *Drosophila* and zebrafish, and may further improve non-imaging techniques such as laser ablation of cells^49^ and microinjection of DNA^50^ in which recovery of healthy organisms post-immobilization is essential.

## Methods

### Strains and culture

Nematode strains were grown on NGM plates seeded with OP50 bacteria. *C. elegans* were imaged as young adults and synchronized by picking L4 stage worms 24 h prior the experiment and transferring them to seeded plates. Alternatively, L1 larval stage animals were picked individually from mixed-stage plates. The following strains were used: QW1217 (*zfIs124*[P*rgef-1*::GCaMP6S]; *ofls355*[P*rab-3*::NLSRFP]), with pan-neuronal expression of nuclear-localized GCaMP and mCherry^48^, and CX16573 (*kyIs587*[P*gpa-6*::GCaMP2.2b, P*unc-122*::dsRed]; *kyEx5662* [P*odr-7*::Chrimson::SL2::mCherry, P*elt-2*::mCherry]), which expresses the Chrimson ion channel and GCaMP calcium indicator in the AWA neuron pair^51^. For optogenetic stimulation, L4 stage animals were transferred to agar plates containing 50 μM all trans-retinal (Sigma-Aldrich) overnight.

*I. paradoxus* pygmy squid adults were collected from sea grass beds in Nagoya, Japan and shipped to the Marine Biological Laboratory (Woods Hole, United States), where they were maintained in aquaria for several months before dying of natural causes. Mature animals readily mated and laid egg masses, with embryos hatching after 1 week to produce actively swimming and hunting squid larvae. While invertebrate care is not regulated under the US Animal Welfare Act, care and use of *I. paradoxus* in this work followed its tenets, and adhered to EU regulations and guidelines on the care and use of cephalopods in research.

### Preparation of materials for hydrogel embedding

PEG hydrogel solutions were prepared by combining 10–20% w/v poly(ethylene glycol) diacrylate (PEG-DA, 3350 MW, 94.45% acrylation, ESI BIO) with 0.05%–0.10% w/v Irgacure 2959 photoinitiator (2-hydroxy-4′-(2-hydroxyethoxy)-2-methylpropiophenone, I2959, BASF) in deionized water (diH_2_O) or 1× S-basal buffer (100 mM NaCl, 50 mM KPO_4_ buffer, pH 6.0). A clean 1″ × 3″ glass slide (VWR Micro Slides) was rendered permanently hydrophobic byexposure to vapors of (tridecafluoro-1,1,2,2-tetrahydrooctyl) trichlorosilane (Gelest) under vacuum for 1 h, or temporarily hydrophobic by wiping with Rain-X Glass Water Repellent. Glass slides were cleaned with ethanol and water, then dried by air gun. For covalent attachment of hydrogels to glass, #1.5 cover slips (Thermo Scientific) were silanized by coating with 3-(trimethoxysilyl)propyl methacrylate (Sigma-Aldrich), 21 mM in ethanol for 3 min, followed by ethanol wash, water rinse, and air dry. Treated glass slides can be prepared months in advance. Spacers were prepared by casting polydimethylsiloxane (PDMS, Sylgard 184, Ellsworth Adhesives) in a 1:10 (curing agent:base) ratio to thicknesses of 100, 200, and 500 µm.

### Embedding live animals in PEG hydrogel

A small volume (1–20 µL) of PEG hydrogel solution with photoinitiator was pipetted onto a hydrophobic glass slide flanked by two PDMS spacers whose thickness matched the desired hydrogel thickness. Animals were transferred into the hydrogel solution by worm pick and optionally cooled on ice or in a freezer to slow animal movement. A coverslip, untreated or silanized, was placed over the hydrogel droplet and supported by the spacers. The glass slide/coverslip sandwich was then placed over a UV light source and illuminated until gelation, 5–100 s (depending on lamp power and hydrogel concentration). The sample was either observed immediately or the hydrogel disk was exposed by lifting the coverslip and adding a drop of aqueous solution over the disk. Hydrogel disks could be transferred to wet agar dishes to keep embedded animals hydrated.

### Mounting *C. elegans* for microscopic imaging

For hydrogel encapsulation, 5 µL of a 10% PEG hydrogel with 0.1% I2959 containing late L1/L2 stage animals was placed on a fluorinated glass slide with 50 µm thick tape spacers, polymerized with 312 nm UV light, and imaged on an upright Zeiss Axioskop microscope with a ×100/1.4 NA objective using DIC optics. Images were captured every hour for 12 h without movement of the sample. For comparison, animals were mounted onto conventional agarose pads with chemical or physical restraint. In azide paralysis conditions, 1–5 µL drop of S-Basal buffer with 25 mM sodium azide was placed on a 1% agarose pad that also contained azide. Animals were picked into this droplet and a coverslip was placed on top before imaging. Alternatively, a 0.25–0.5 µL drop of polystyrene nanobeads (100 nm, Polysciences, Inc.) was placed on a 10% agarose pad prior to adding animals, a coverslip, and imaging^5^.

### Recovery of *C. elegans* from PEG hydrogels

Young adult worms were embedded in 5 µL 20% PEG hydrogels with 0.1% I2959 by crosslinking for 15 s at 312 nm on an unsilanized glass slide with 100 µm tape spacers. Hydrogels were transferred to an agar plate to keep hydrated and were stored at room temperature (22–23 °C). After 24 h, hydrogels were separated with tweezers and animals were scored for viability (movement and pharyngeal pumping, noting any signs of mechanical damage or internal egg hatching). Some animals were transferred to cover slips with 1% agarose pads for imaging.

### Characterization of illumination and crosslinking conditions

Several UV light sources were used for crosslinking the hydrogel: a gel box transilluminator at 308 nm (Hoefer Scientific Instruments, model UVTM-25) and two handheld compact UV transilluminators, at 312 nm (International Biotechnologies, Inc, model UVH, 12 W) and at 365 nm (UVP, model UVGL-15, 4 W). Light power was measured with a power meter (Thorlabs PM100) and 200–1100 nm sensor (Thorlabs S120UV) placed directly on the light source or on a glass slide or coverslip. Power values were converted to irradiance by dividing by the area of the 9.5 mm diameter sensor. Illumination spectra were obtained using a spectrometer (mut GmbH TRISTAN) with a fiber light guide (200–1100 nm range, 400 µm diameter). The photoinitiator absorbance was obtained with a spectrophotometer (Thermo Scientific Multiskan Spectrum).

The hydrogel crosslinking time was determined optically by monitoring movement of young adult worms during crosslinking. Gels of varying PEG-DA concentration, photoinitiator concentration, and geometry (spacer thickness and volume) were compared for each light source. Videos were captured at 1 frame per second (fps) using a Leica S6D stereoscope, converted to reflectance illumination by replacing one eyepiece with a white LED lamp, and recording via the opposite eye path with a UniBrain Fire-I 580c camera. Animal movement was analyzed by comparing frame-to-frame image differences in ImageJ. Crosslinking time was determined as the time until image difference measurements reduced to within 1 standard deviation of noise, and verified visually during observation of videos.

### Quantification of movement of hydrogel-embedded animals

Young adult QW1217 worms with pan-neuronal expression of nuclear-localized mCherry were embedded in 10 and 20% PEG hydrogels (3 µL with 100 µm spacer, 0.1% I2959, 20 s exposure using a 312 nm UV source). Worms received either no pretreatment, exposure to hypo-osmotic (diH_2_O, 0 mOsm) or hyper-osmotic buffers (0.5 M glycerol in diH_2_O, 500 mOsm or 1.5× S-Basal, 420 mOsm) for 10 min, or cooling in a –20 °C freezer or in contact with ice for 1–3 min prior to crosslinking. PEG hydrogel solutions were prepared in diH_2_O or S-Basal buffer, and some contained the paralytic reagents 25 mM sodium azide^52^ or 1 mM tetramisole hydrochloride (Sigma)^15^. Other hydrogel solutions were prepared in 500 mM glycerol or 1.5× S-Basal hyper-osmotic buffers. After crosslinking, all hydrogels were submerged in an aqueous solution of either S-Basal, diH_2_0, sodium azide (25 mM) or tetramisole (1 mM) for imaging. Videos were captured with a Hamamatsu Orca-Flash 4.0 camera at 1 fps for 3 min on a Zeiss AxioObserver inverted epifluorescence microscope with a ×20/0.5 NA objective.

Animal movement was analyzed by comparing frame-to-frame image differences (Supplementary Fig. 4). First, the absolute value differences between each pair of consecutive frame averaged pixel intensities were calculated. Next, the difference image stack was smoothed (average of its 3 × 3 neighborhood). To reduce the contribution of pixel noise, a value of 10 (corresponding to average pixel noise) was subtracted uniformly from each frame and negative values were set to 0. Regions of Interest (ROIs) were selected over the head (nerve ring) and ventral cord and a background region (BG). The MI was calculated as ${\mathrm {MI}}\left( {fr} \right) = \overline {\Delta I} _{{\mathrm{ROI}}}\left( {fr} \right)/\left( {\bar I_{{\mathrm{ROI}}} - \bar I_{{\mathrm{BG}}}} \right)$, where $\overline {\Delta I} _{{\mathrm{ROI}}}$ is the mean of difference images across the ROI, and $\bar I_{{\mathrm{ROI}}}$ and $\bar I_{{\mathrm{BG}}}$ are the mean intensity of the ROI and background regions for the first frame, respectively. Here, identical sequential frames would have a MI of zero, whereas images that have changes in position, rotation, focus, or intensity have increasing movement index values.

Movement in the image plane was quantified by tracking cell nucleus position over 3 min. Individual neurons were tracked using NeuroTracker^15^ and centroid positions were used to determine the range of axial movement by the animal.

### Measurement of PEG and agarose hydrogel optical resolution

Fluorescent beads (125 nm, Zebrite Highlighter, Zebra) were embedded in 100 μm thick hydrogels composed of 10%, 13%, and 20% PEG-DA or 1% low-melt agarose (APEX, Genesee Scientific). PEG hydrogels were crosslinked at 312 nm for 30 s, as described above, and LMA hydrogels were prepared by sandwiching a drop of melted agarose with beads between a glass slide and coverslip with a 100 μm spacer and allowing 1–2 min for cooling. All gels were washed with diH_2_O prior to scanning volumetric image slices with a laser scanning confocal microscope (Leica TCS SP5) at 488 nm (27% laser power) with a ×63/ 1.4 NA objective. About 130 z-slices with 40 nm spacing were captures for a 15.4 × 15.4 × 5.2 mm^3^ field of view. Point spread functions were obtained in ImageJ by reconstructing the spatially-calibrated volume and extracting 2D slices (*xy*, *yz*, *xz*) centered on selected beads, averaging 5 pixels wide. Full-width half-maximum measurements were calculated using MATLAB. Bead sizes were confirmed with a Malvern Zetasizer (mean 126 ± 26 nm s.d.).

### Measurement of PEG and agarose autofluorescence

Fluorescence spectra were measured for PEG-DA (20% with 0.1% I2959) and 1% low-melt agarose using a Hitachi F-4500 fluorescence spectrophotometer and quartz cuvette. Excitation and emission wavelengths were scanned from 200–900 nm at 10 nm intervals with a 5 nm slit.

### Light-sheet imaging of optogenetically-stimulated worms

Young adult animals co-expressing Chrimson and GCaMP2.2b in the AWA chemosensory neurons were embedded in a PEG hydrogel bonded to a 24 × 50 mm methacrylate-silane-treated coverslip. Animals were picked into a 2.4 µL drop of 13.3% PEG-DA solution with 0.067% I2959 in 500 mM glycerol in diH_2_O. After 5 min, the sample was cooled on ice for 30 s and exposed to UV light for 30 s with a 308 nm handheld lamp. Cover slips were mounted into a light-sheet chamber (ASI, I-3078-2450) and filled with ~5 mL diH_2_O. The diSPIM recorded the calcium response of AWA with a 488 nm excitation laser (Vortran Stradus VersaLase) at 1 mW power setting and a 525/50 nm emission filter. Single-view volumetric stacks (40 slices with 1 µm spacing, 166 × 166 × 40 µm^3^), were obtained at 1 volume per second for three 60 min recording sessions beginning at *t* = 0 h, 6 h, and 13.5 h, for a total of 432,000 image frames. A red LED light (617 nm, Mightex, with 620/30 nm filter) was mounted either above the stage, illuminating the animals at a 45° angle, or from below with a 600 nm shortpass dichroic through a ×4 objective, and controlled via MATLAB and an Arduino controller. Red light pulses, 10 s in duration, were repeated each minute during recordings. For each time point, the volume stack was compressed into a single maximum projection plane, and intensity values were integrated across ROIs surrounding each neuron or neuronal process. After subtracting background intensity from a nearby region, fluorescence intensity (*F*) integrated across the neuron or neurite ROI was normalized to the initial intensity averaged over 1 s (*F*_*0*_). No interference was observed in background or neural ROIs during red light exposure.

### Light-sheet imaging of living pygmy squid

Pygmy squid (*Idiosepius paradoxus*) at three days post-hatching were treated with 1 µM BODIPY 564/570 C_3_ succinimidyl ester vital dye (ThermoFisher D2222) in filtered seawater for 1 h to label cell boundaries and generally visualize morphology, modifying methods for the marine worm *Platynereis dumerelli*^53^. Squid were washed five times in filtered seawater, then paralyzed by exposure to 3.75% MgCl_2_ in seawater for 15 min and transferred to a hydrophobic glass slide. Excess seawater was aspirated by pipette such that ~4 µL liquid remained. To this, 16 µL of 20% PEG-DA in seawater with photoinitiator was added and gently mixed. A methacrylate-silane-treated 24 × 50 mm^2^ coverslip was placed 1.2 mm over the droplet using glass slides as spacers. The hydrogel was crosslinked by exposure to 365 nm UV light for 1 minute and remained adhered to the coverslip. The hydrogel was rinsed with fresh seawater to wash out the anesthetic and the coverslip was mounted into the light-sheet chamber which was then filled with seawater. Dual-view volumetric stacks (30 slices with 1 µm spacing, 332 × 332 × 30 µm^3^) were obtained using a 561 nm laser (4 mW power setting).

### Statistical analysis

Statistical comparisons were made by two-way ANOVA with significance level set at *α* = 0.05, followed by Bonferroni’s multiple comparison tests. Data are presented as mean ± standard deviation, unless otherwise noted.

### Data availability

The datasets generated during and/or analyzed during the current study are available from the corresponding author on reasonable request.

## Electronic supplementary material

Supplementary Information

Description of additional supplementary information

Supplementary Movie 1

Supplementary Movie 2
