# Supplementary material for: Rapid and gentle hydrogel encapsulation of living organisms enables long-term microscopy over multiple hours
Source: Commun Biol. 2018 Jun 21;1:73. doi: 10.1038/s42003-018-0079-6 (PMC6123791; doi:10.1038/s42003-018-0079-6)
Supplement: Supplementary file 1 — Supplementary Information [file 42003_2018_79_MOESM1_ESM.pdf]

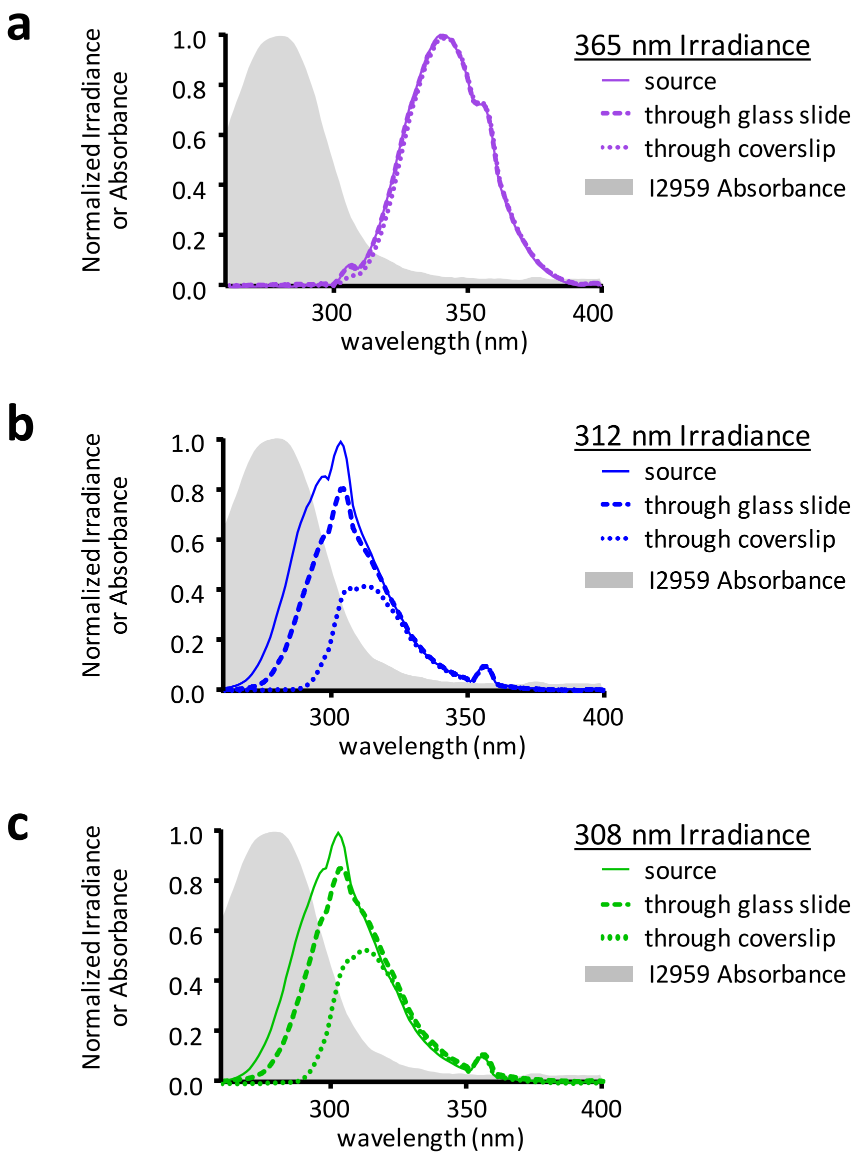

Supplementary Figure 1. Irradiance of different ultraviolet light sources through different substrates. Normalized absorbance spectrum of Irgacure 2959 photoinitiator and irradiance spectra of **(a)** 365 nm, **(b)** 312 nm, and **(c)** 308 nm UV exposure sources alone, through a 1 mm thick glass slide, and through a #1.5 coverslip. Irradiance curves for each substrate were normalized to the irradiance of each source at 365 nm. Greater overlap between UV emission and photoinitiator absorbance accelerates hydrogel crosslinking (Fig. 2).

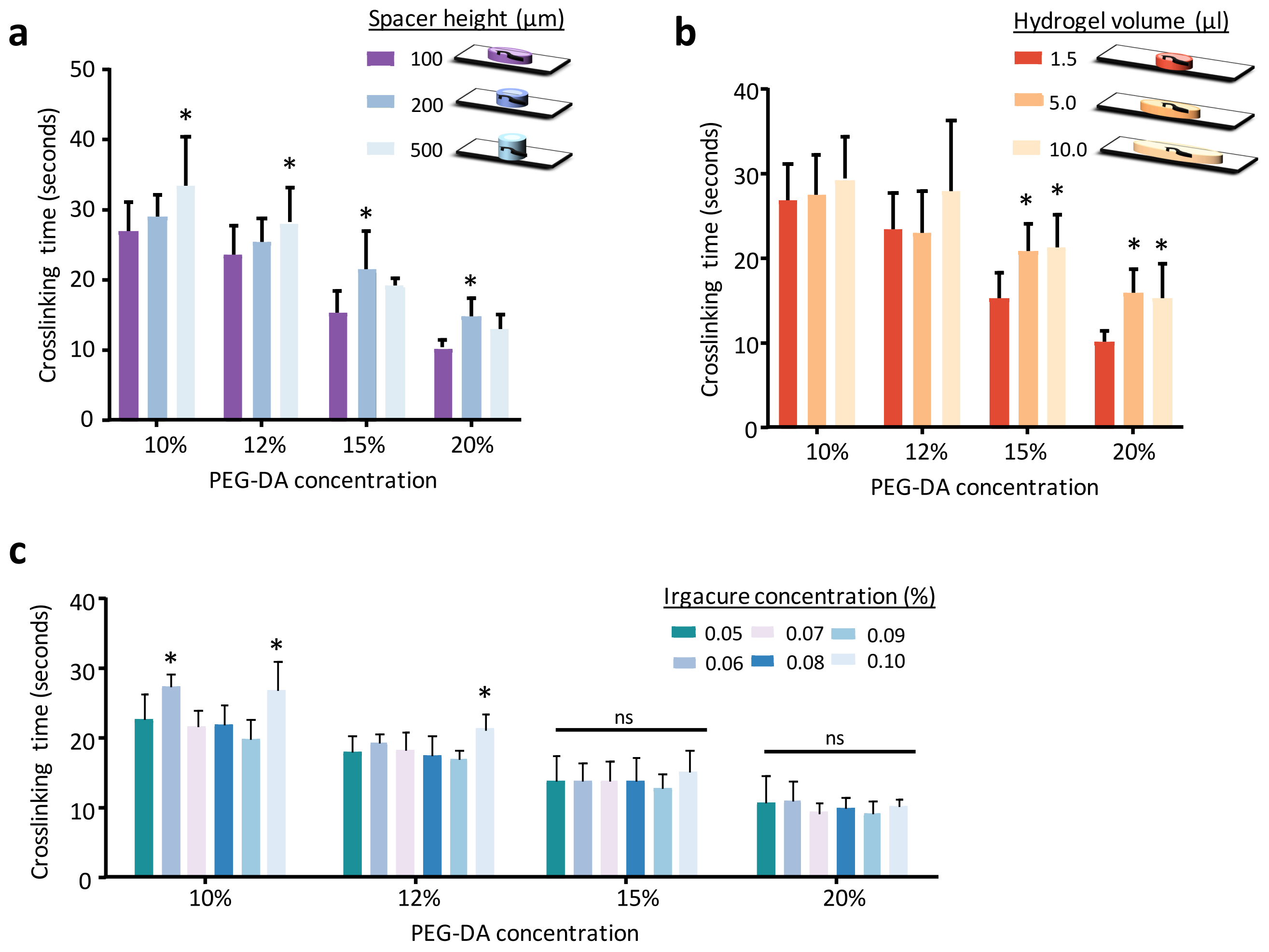

Supplementary Figure 2. Characterization of PEG hydrogel crosslinking times for different PEG-DA concentrations and geometries. Each bar represents the average amount of time required to immobilize living wild-type young adult *C. elegans*, a practical measure of hydrogel crosslinking rate. Various hydrogel concentrations, volumes, heights, and I2959 photoinitiator concentrations were exposed with a 312 nm UV source. **(a)** Spacer height from 100 to 500 µm had minimal effect on crosslinking a 1.5 µL volume with 0.10% I2959. **(b)** Increasing hydrogel volume from 1.5 to 10 µL slowed crosslinking, with 100 µm spacer and 0.10% I2959. **(c)** Irgacure 2959 photoinitiator concentration from 0.05% to 0.1% had minimal effect on crosslinking a 1.5 µL volume with 100 µm spacer. Each bar represents n = 10 trials, with each trial averaging immobilization times from 2 - 5 worms. Bars represent mean and standard deviation. Statistics were performed using ordinary 2-way ANOVA with Bonferroni's *post hoc* tests for pairwise comparisons, *$P < 0.05$ compared to 100 µm (a), 1.5 µL (b) and 0.05% (c) conditions.

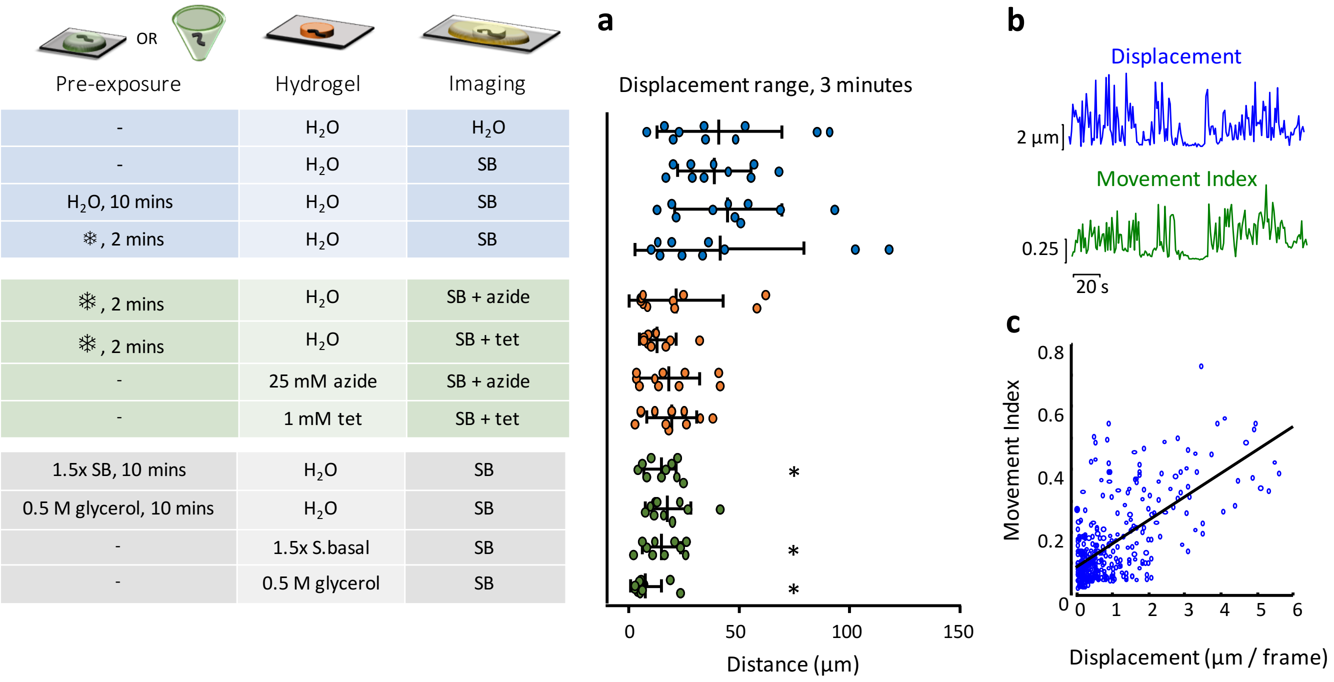

Pre-exposure	Hydrogel	Imaging
-	H_2O	H_2O
-	H_2O	SB
H_2O, 10 mins	H_2O	SB
❄, 2 mins	H_2O	SB
❄, 2 mins	H_2O	SB + azide
❄, 2 mins	H_2O	SB + tet
-	25 mM azide	SB + azide
-	1 mM tet	SB + tet
1.5x SB, 10 mins	H_2O	SB
0.5 M glycerol, 10 mins	H_2O	SB
-	1.5x S.basal	SB
-	0.5 M glycerol	SB

Supplementary Figure 3. Movement of hydrogel-embedded animals depends on conditions before, during, and after hydrogel crosslinking. Pre-exposure to hypo- or hyper-osmotic solutions for 10 minutes, or cooling pretreatment (❄ on ice or in a -20°C freezer for 2 minutes) occurred in a droplet or microtube. Hydrogel solutions were prepared in water (H_2O), S-Basal buffer (SB), 25 mM sodium azide (azide) or 1 mM tetramisole (tet) in water, 500 mM glycerol in water, or 1.5x S-Basal buffer. **(a)** Mean displacement range over 3 min, from n = 7 - 10 worms. Vertical line and error bars represent mean and standard deviation. **(b)** Comparisons between displacement and movement index are shown for one representative worm. **(c)** Correlation of movement index versus displacement, $R^2 = 0.45$. Statistics were performed using ordinary 2-way ANOVA with Bonferroni's *post hoc* tests for pairwise comparisons: $*P < 0.05$ is compared with the hydrogel control with S-Basal solution.

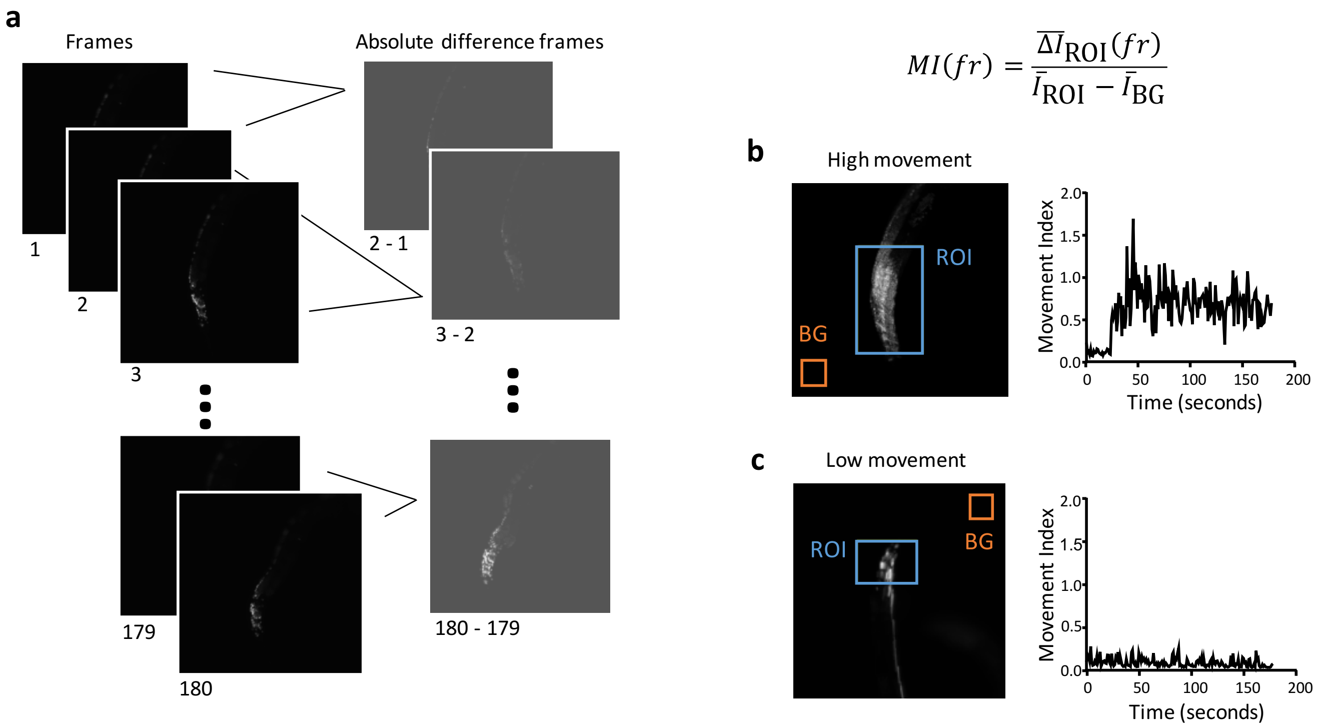

Supplementary Figure 4. Movement index calculations. Movement index (*MI*) is calculated per frame as the absolute difference in pixel intensities between consecutive frames **(a)**, averaged across a region of interest (ROI) and normalized by dividing by the average intensity in the ROI minus background intensity from the first frame. High-movement **(b)** and low-movement **(c)** examples show the time course of instantaneous movement over 180 frames at 1 fps.

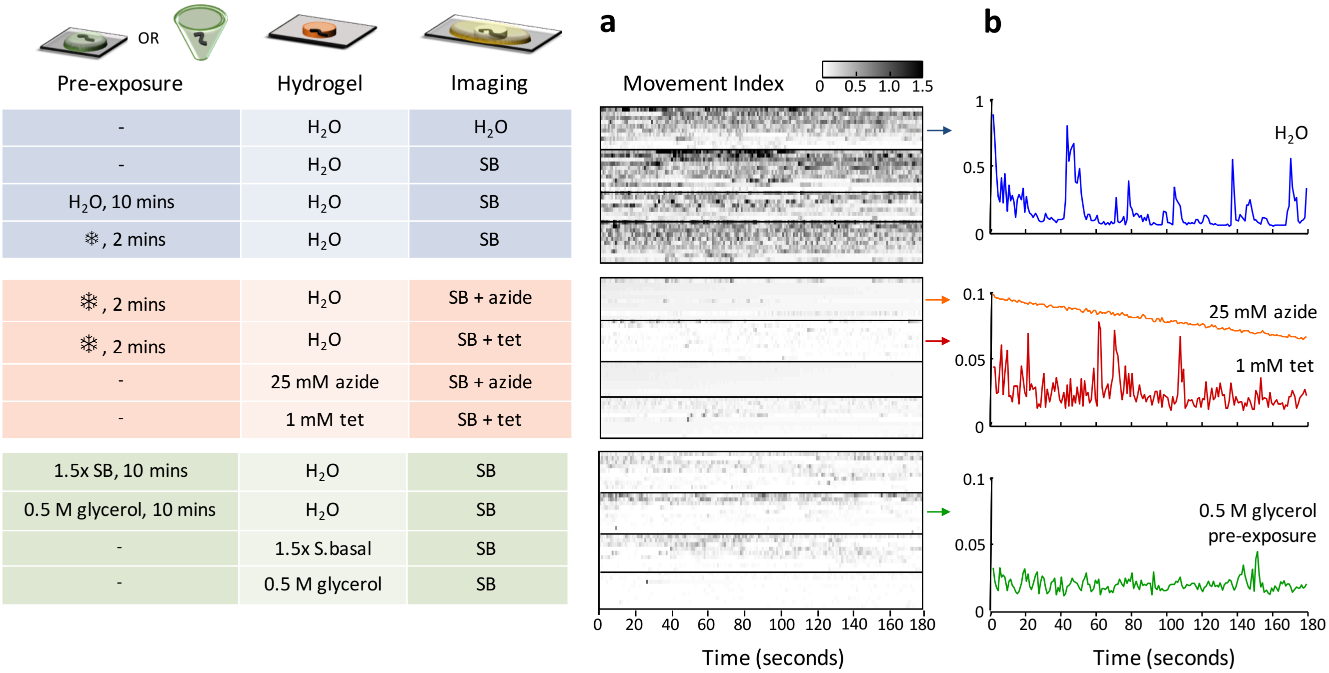

Supplementary Figure 5. **Movement dynamics of young adult animals encapsulated using different hydrogel crosslinking conditions.** Pre-exposure to hypo- or hyper-osmotic solutions for 10 minutes, or cooling pretreatment (❄ on ice or in a -20°C freezer for 2 minutes) occurred in a droplet or microtube. Hydrogel solutions were prepared in water (H_2O), S-Basal buffer (SB), 25 mM sodium azide (azide) or 1 mM tetramisole (tet) in water, 500 mM glycerol in water, or 1.5x S-Basal buffer.

(**a**) Heat maps show movement index vs. time for the 3-minute duration of fluorescent imaging. Each row represents the movement index of an individual animal.

(b) Movement indices for selected treatment groups (arrows), showing one animal each displaying median movement per group.

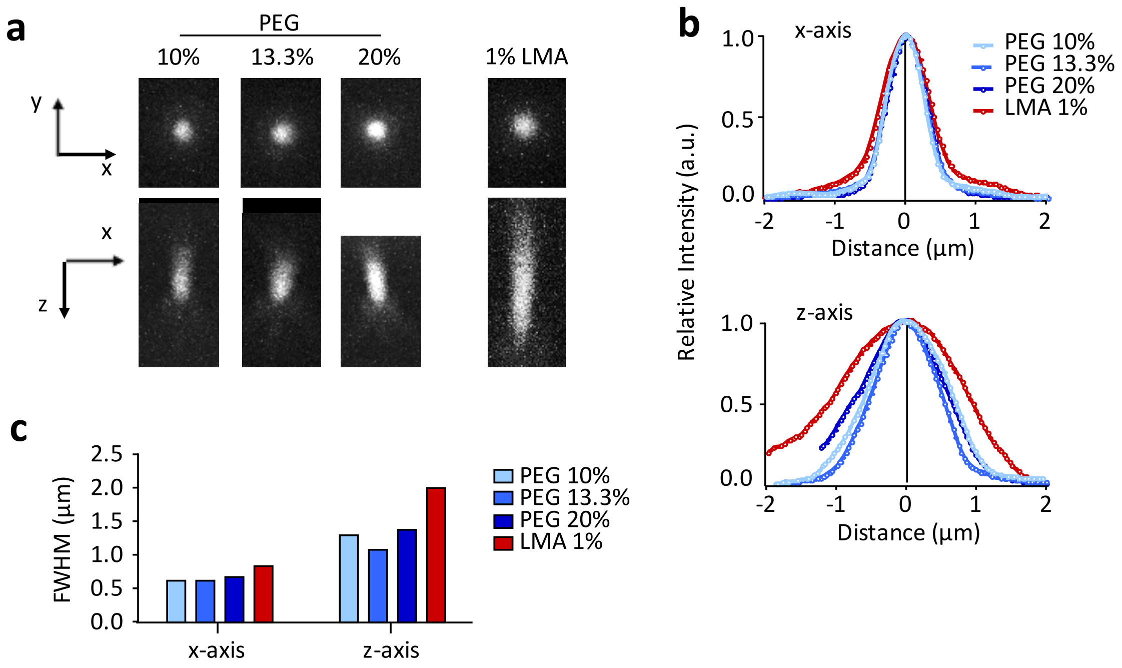

Supplementary Figure 6. Comparison of optical resolution in different hydrogels. Point spread functions of embedded fluorescent nanobeads were measured in 10%, 13.3% and 20% PEG hydrogels versus 1% low melt agarose (LMA).

(a) XY and XZ maximum projections of ~125-nm fluorescent beads. Scale bar (arrows), 1 μm.

(b) Relative intensity line scans along the center of fluorescent beads in each hydrogel in the x- and z-dimensions.

(c) Quantification of full width at half maximum (FWHM) of intensity profiles from panel b. Data are from one representative bead in each hydrogel.

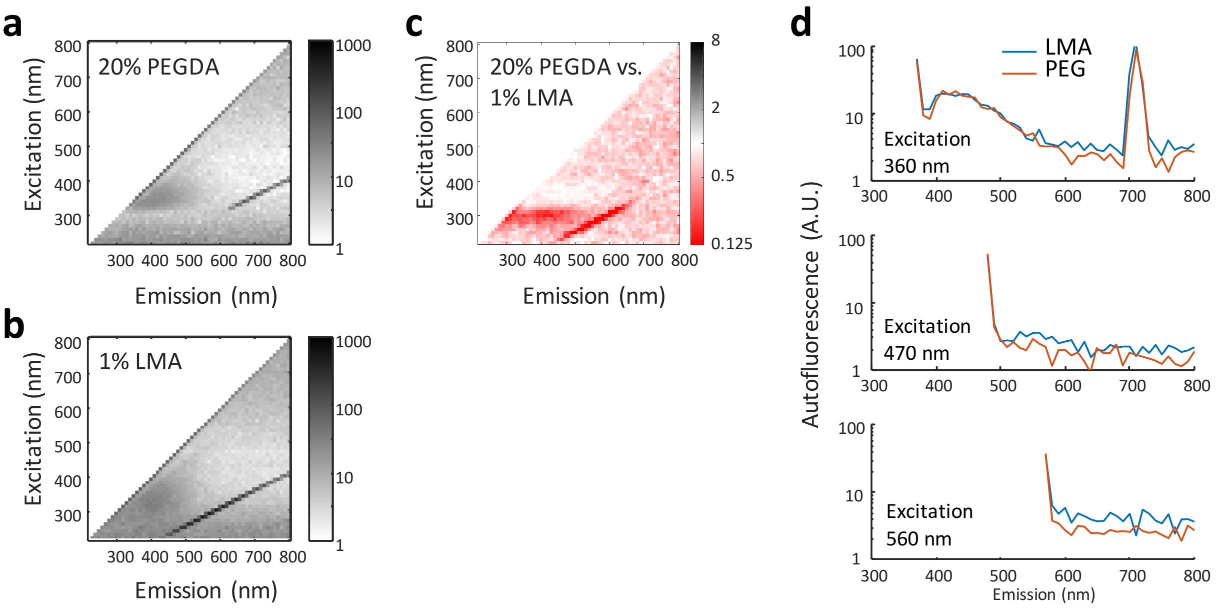

Supplementary Figure 7. Comparison of autofluorescence of PEG hydrogel and agarose. Autofluorescence is slightly lower for PEG hydrogels.

(a) Excitation-emission matrix of relative fluorescence at wavelengths of 200 – 800 nm with a 10 nm and 5 nm slit width for 20% PEGDA.

(b) Excitation-emission matrix of relative fluorescence for 1% low melt agarose (LMA), as in panel b.

(c) Relative autofluorescence of 20% PEGDA vs. 1% LMA. Red colors indicate lower autofluorescence of PEG.

(d) Emission spectra for excitation at common wavelengths for different fluorophores: 360 nm (DAPI), 470 nm (GFP), and 560 nm (RFP).

Peak at twice the excitation wavelength is a measurement artifact.

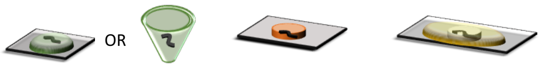

Pre-exposure	Hydrogel	Imaging
-	20% PEG	SB
-	12% PEG	SB
1.5x SB, 10 mins	20% PEG	SB
1.5x SB, 10 mins	12% PEG	SB

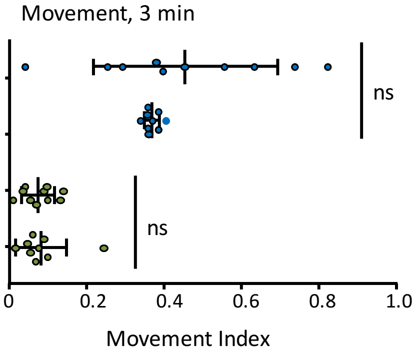

Supplementary Figure 8. Movement of animals encapsulated in 12% and 20% PEG hydrogels. Young adult animals embedded in 20% and 12% PEG-DA hydrogels showed similar movement under control and hyper-osmotic pre-exposure conditions (10 minutes pre-exposure to 1.5x S. Basal buffer in a droplet or microtube). Each dot plot represents the mean movement index over 3 min, from n = 9 - 10 worms. Vertical and error bars represent mean and standard deviation. Statistics were performed using ordinary 2-way ANOVA with Bonferroni's *post hoc* tests for pairwise comparisons. ns, not significant.
