# Supplementary material for: Rapid and gentle hydrogel encapsulation of living organisms enables long-term microscopy over multiple hours
Source: Commun Biol. 2018 Jun 21;1:73. doi: 10.1038/s42003-018-0079-6 (PMC6123791; doi:10.1038/s42003-018-0079-6)
Supplement: Supplementary file 2 — Description of additional supplementary information [file 42003_2018_79_MOESM2_ESM.docx]

**Supplementary Movie 1.** **Crosslinking of *C. elegans* in hydrogel disks.** Video shows young adult animals swimming in polymer solutions of 12%, 15%, and 20% PEG-DA. At 0:06, the 312 nm UV lamp is turned on, and animals become encapsulated several seconds later. Video is accelerated 5x. Note that animals were not cooled prior to gelation, in order to observe movement more easily. Cooling eliminates movement before gelation, allowing positioning of animals within the hydrogel disk.

**Supplementary Movie 2.** **Movement of young adult *C. elegans* embedded in 20% PEG-DA hydrogels.** Animals were imaged in buffer (top), in 1 mM sodium azide (middle), and buffer following pre-exposure to 500 mM glycerol as in Fig. 3. Video is accelerated 3x.
